# ^177^Lu-Labeled Anticlaudin 6 Monoclonal Antibody for Targeted Therapy in Esophageal Cancer

**DOI:** 10.2967/jnumed.124.268487

**Published:** 2025-03

**Authors:** Huan Du, Xiaofei Hao, Binwei Lin, Mingming Tang, Decai Wang, Xia Yang, Jing Wang, Liling Qin, Yuchuan Yang, Xiaobo Du

**Affiliations:** 1 Mianyang Central Hospital, School of Medicine, University of Electronic Science and Technology of China, Mianyang, China;; 2 NHC Key Laboratory of Nuclear Technology Medical Transformation, Mianyang Central Hospital, School of Medicine, University of Electronic Science and Technology of China, Mianyang, China;; 3 Clinical Medical School, North Sichuan Medical College, Nanchong, China;; 4 Sichuan Clinical Research Center for Radiation and Therapy, Mianyang Central Hospital, Mianyang, China;; 5 Institute of Nuclear Physics and Chemistry, China Academy of Engineering Physics, Mianyang, China; and; 6 Pathology Department, First People’s Hospital of Mianyang, Mianyang, China

**Keywords:** CLDN6, ^177^Lu, ^89^Zr, esophageal cancer, targeted radiopharmaceutical therapy

## Abstract

Advanced or metastatic esophageal cancer (EC) is associated with poor prognosis, necessitating new and effective treatment methods. We assess whether claudin 6 (CLDN6) is a useful target for the imaging and radiopharmaceutical therapy of EC using a novel pair of radioactive nuclides, ^89^Zr and ^177^Lu. **Methods:** CLDN6 messenger RNA expression was evaluated in 2 EC datasets (*n* = 436) and through a retrospective analysis of 109 patients with EC. We then used an anti-CLDN6 monoclonal antibody (IMAB027) labeled with ^89^Zr and ^177^Lu ([^89^Zr]Zr-DFO-IMAB027 and [^177^Lu]Lu-DOTA-IMAB027) for PET imaging and therapy, respectively. Imaging and biodistribution analyses were performed using the TE-1-CLDN6 xenograft model. Finally, the therapeutic potential of [^177^Lu]Lu-DOTA-IMAB027 was evaluated in both the TE-1-CLDN6 and the CLDN6-PDX (patient-derived xenograft) models. **Results:** CLDN6 messenger RNA expression was elevated in EC compared with healthy esophageal tissues. The CLDN6 expression rate was 0 in healthy esophageal tissue but was 79.8% in EC tissue. The [^89^Zr]Zr-DFO-IMAB027 showed the ability to effectively image EC xenografts with high CLDN6 expression. In the TE-1-CLDN6 model, there was a significant difference in tumor volume between the 11.1-MBq [^177^Lu]Lu-DOTA-IMAB027 treatment group and the control group (*P* < 0.001). The tumor growth inhibition rate in the 11.1-MBq [^177^Lu]Lu-DOTA-IMAB027 group was 101.74%. In the PDX model, significant differences in tumor volume were observed among all [^177^Lu]Lu-DOTA-IMAB027 treatment groups and the control group (*P* < 0.05). Specifically, the tumor growth inhibition rate of the 11.1-MBq [^177^Lu]Lu-DOTA-IMAB027 group was 79.04%, whereas that of the 3.7-MBq group was 77.20%. However, the difference in efficacy between the high-dose and low-dose groups was not statistically significant (*P* > 0.05). **Conclusion:** The differential expression of CLDN6 between tumors and the normal esophagus shows its potential as a diagnostic and therapeutic target for EC. The radiotracer [^89^Zr]Zr-DFO-IMAB027 showed high contrast when visualizing CLDN6-expressing xenografts for PET imaging, and [^177^Lu]Lu-DOTA-IMAB027 induced rapid tumor regression in both the TE-1-CLDN6 and the CLDN6-PDX models. This research has implications for improving the radioligand diagnosis and treatment of EC.

## Visual Abstract

**Figure:**
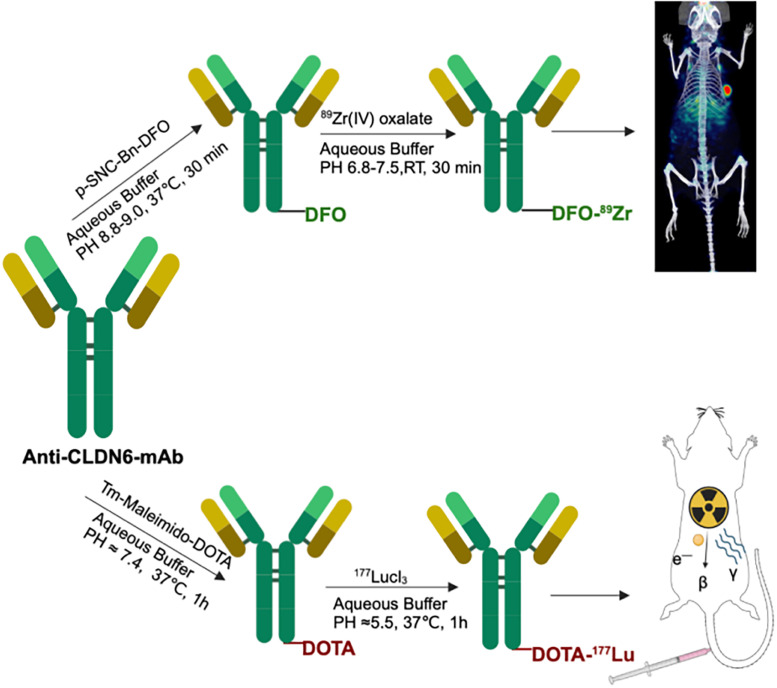

Esophageal cancer (EC), characterized by rapid progression and propensity for early metastasis, is the sixth leading cause of cancer-related mortality worldwide (1). Approximately 22,370 new cases of EC were predicted in 2024 in the United States, with an estimated 16,130 fatalities (2). Despite advances in surgical techniques, chemotherapy, and radiation therapy, the 5-y survival rate of patients with advanced EC remains below 20% (3,4). This highlights the pressing need for innovative therapeutic strategies capable of targeting the molecular attributes of cancer cells with minimal impact on normal tissues.

Targeted radiopharmaceutical therapy (TRT) is an emerging form of precision cancer therapy based on the combination of targeted carriers and radioactive isotopes (5). The advantage of TRT lies in its ability to selectively irradiate primary and metastatic lesions while minimizing damage to surrounding normal tissues (6). [^177^Lu]Lu-prostate-specific membrane antigen and [^177^Lu]Lu-DOTATATE have been approved by the U.S. Food and Drug Administration for the routine treatment of patients with metastatic prostate and neuroendocrine cancer (7,8). To the best of our knowledge, no studies have been conducted on the application of TRT for the treatment and diagnosis of EC (9).

Claudins, a family of integral membrane proteins, are key components of tight junctions that play critical roles in maintaining cell polarity and regulating epithelial barrier permeability (10,11). Claudin 6 (CLDN6), a member of the claudin family, is important for maintaining normal cell function (12). CLDN6 only exists in human embryonic cells and is not expressed in normal adult tissues (13); however, it is specifically expressed in various tumor tissues, such as gastric cancer, ovarian cancer, colon cancer, and germ cell carcinoma (14–18). In ovarian cancer, CLDN6 is highly expressed and actively promotes tumor cell proliferation (17). Similarly, in endometrial cancer, its overexpression is associated with tumor cell growth and survival, potentially through activation of the phosphatidylinositol 3-kinase/protein kinase B signaling pathway (14). In hepatocellular carcinoma, CLDN6 facilitates epithelial–mesenchymal transition, thereby enhancing the proliferative and invasive capabilities of liver cancer cells (19). In germ cell tumors, CLDN6 demonstrates highly specific expression and is strongly implicated in promoting tumor cell proliferation (20). Additionally, studies have shown that CLDN6 is expressed in esophageal adenocarcinoma. Its protein expression has been identified as an independent unfavorable prognostic factor in esophageal adenocarcinoma, associated with shorter overall survival (21). These findings highlight CLDN6 as a critical molecule in cancer biology, with potential relevance to EC and its prognosis. Given its differential expression between tumor and normal tissues, CLDN6 is considered a promising therapeutic target for TRT.

IMAB027, a humanized monoclonal antibody targeting CLDN6, has been evaluated in clinical trials for ovarian cancer (NCT02054351) and refractory germ cell tumors (NCT03760081). However, IMAB027 monotherapy has shown limited efficacy in germ cell tumors (22). Currently, anti-CLDN6 monoclonal antibodies have been used to synthesize antibody–drug conjugates, which have demonstrated robust tumor regression in ovarian and endometrial cancers (6). Nevertheless, to the best of our knowledge, no studies to date have investigated the use of CLDN6-targeted TRT for the treatment of solid tumors. This study aims to elucidate the expression of CLDN6 in normal esophageal and EC tissues and evaluate its potential as a therapeutic target for the diagnosis and treatment of CLDN6-expressing EC.

## MATERIALS AND METHODS

Details of flow cytometry, cell lines, cell transfection, chemical synthesis, radiolabeling methods, binding, block assays, immunohistochemistry, immunofluorescence, PET/CT, biodistribution, and radiopharmaceutical therapy are provided in the supplemental materials (available at http://jnm.snmjournals.org).

### Immunohistochemistry Analysis of EC Biopsy Samples

In total, 109 eligible EC specimens and 16 normal esophageal specimens were collected from Mianyang Central Hospital (Mianyang, China); CLDN6 expression was measured using immunohistochemistry. Immunohistochemistry scoring criteria were based on published literature (16). The Ethics Committee of Mianyang Central Hospital approved this retrospective study and animal experiment, and the requirement to obtain informed consent was waived (S-2020-016, S20230203).

### Statistical Analysis

Statistical analyses were performed using IBM’s SPSS Statistics for Windows software (version 22.0). Binary comparisons between the 2 treatment arms were performed using unpaired 2-tailed Student *t* tests. Differences at the 95% confidence level (*P* < 0.05) were considered statistically significant.

## RESULTS

### Expression of CLDN6 in EC

**FIGURE 1.**
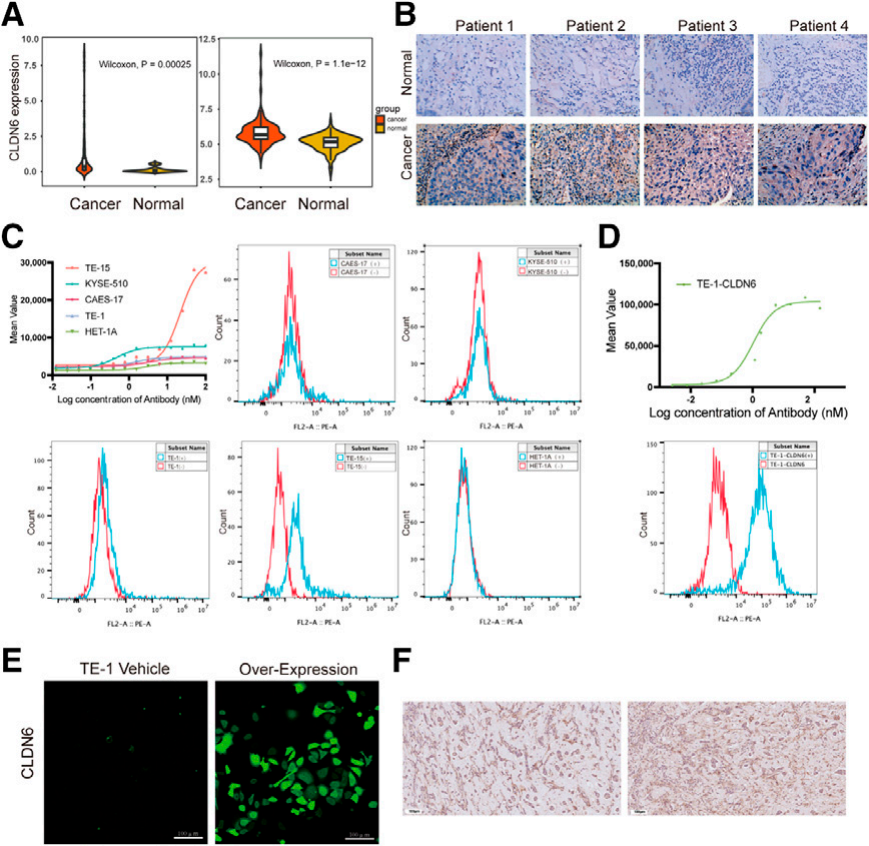
(A) Violin plot showing CLDN6 messenger RNA expression levels in EC and normal esophageal tissues from Cancer Genome Atlas and GSE53624 cohorts. (B) Immunohistochemistry staining of CLDN6 in representative normal esophageal and EC tissues (×40 magnification). (C and D) Flow cytometry was used to detect CLDN6 expression in KYSE-510, TE-1, TE-15, CAES-17, HET-1A, and TE-1-CLDN6 cells. (E) TE-1 cells and TE-1-CLDN6 green fluorescent protein cells exhibiting stable CLDN6 expression under confocal microscopy. (F) CLDN6 immunohistochemistry images of TE-1-CLDN6 tumor model. FL2-A = fluorescence intensity of second fluorescence channel; PE-A = fluorescence intensity of the phycoerythrin dye.

The results of the 2 cohorts showed that the median CLDN6 messenger RNA expression was elevated in EC relative to that in healthy esophageal tissue (*P* < 0.001; Fig. 1A). The immunohistochemistry results showed that the expression level of CLDN6 in EC was significantly higher than that in healthy esophageal tissue (79.8% vs. 0%; *P* < 0.001). Among 109 EC specimens, 87 expressed CLDN6 and 22 did not, indicating a positivity rate of 79.8%. Among samples with CLDN6-positive expression, 50 were +, 25 were ++, and 12 were +++. No CLDN6 expression was observed in any of the 16 normal esophageal samples, with a positive expression rate of 0 (Fig. 1B).

### Cellular Screening and Cell Transfection

Flow cytometry analysis of EC cell lines revealed low levels of CLDN6 expression in TE-1, CAES-17, and KYSE-510 cells. TE-15 exhibited moderate levels of CLDN6 expression, whereas the normal esophageal epithelial cell line HET-1A showed almost no CLDN6 expression (Fig. 1C). Since no EC cell lines with high CLDN6 expression were identified, we used lentiviral transfection of green fluorescent protein–tagged CLDN6 into TE-1 cells to establish a stable EC cell line with high CLDN6 expression, referred to as TE-1-CLDN6. Stable and high expression of CLDN6 in TE-1-CLDN6 cells was confirmed through flow cytometry and immunofluorescence analysis. Finally, TE-1-CLDN6 cells were used to establish a subcutaneous positive EC xenograft model, and immunohistochemistry validated the high expression of CLDN6 in the TE-1-CLDN6 model (Figs. 1D and 1F).

### Synthesis and Radiolabeling

**FIGURE 2.**
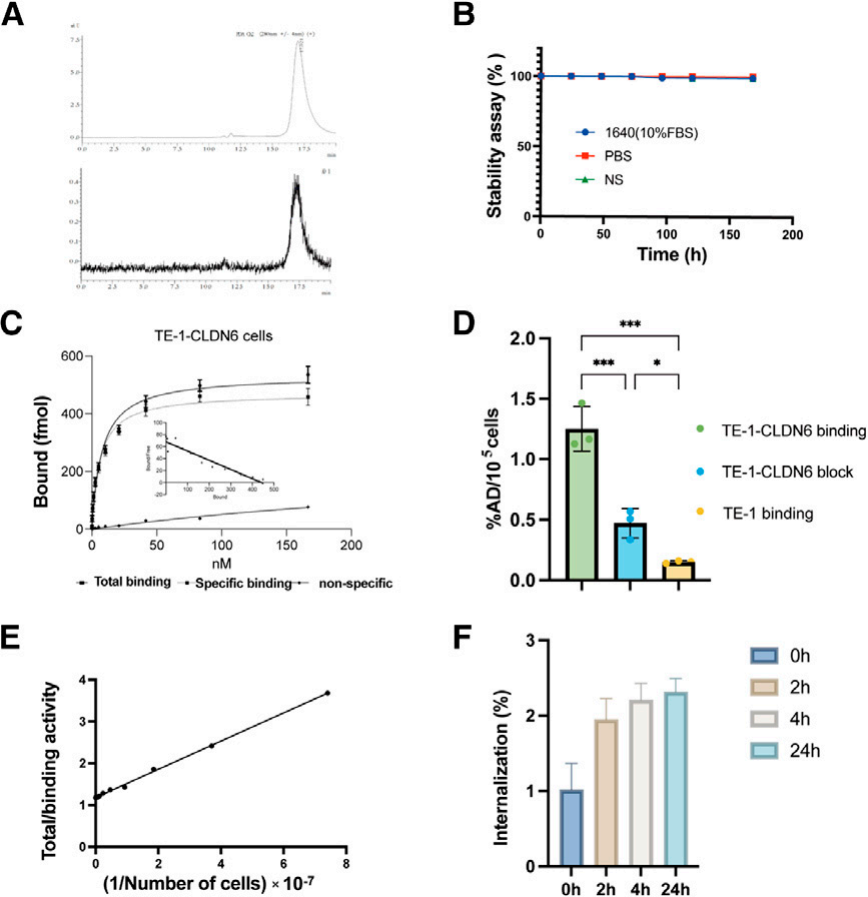
(A) Radiochemical purity of [^177^Lu]Lu-DOTA-IMAB027 determined by high-performance liquid chromatography was >99%. (B) Stability of [^177^Lu]Lu-DOTA-IMAB027 detected by thin-layer chromatography in NS, phosphate buffered saline (PBS), and RPMI 1640 (1640) (10% fetal bovine serum). (C) Specific binding of [^177^Lu]Lu-DOTA-IMAB027 in TE-1-CLDN6 cells. (D) Blocking experiment to detect radioactive uptake of TE-1-CLDN6/TE-1 in response to [^177^Lu]Lu-DOTA-IMAB027 and examine radioactive uptake of TE-1-CLDN6 when exposed to excessive amounts of IMAB27 and [^177^Lu]Lu-DOTA-IMAB027. (E) Immunoactivity fraction of [^177^Lu]Lu-DOTA-IMAB027 in TE-1-CLDN6 cells. (F) Internalization of [^177^Lu]Lu-DOTA-IMAB027 in TE-1-CLDN6 cells. %AD = percent added dose.

IMAB027 was first conjugated with p-NCS-Bz-DFO (C121; Ruixibio) or DOTA-maleimide (DPG-6350; About bioleaf) and then labeled with ^89^Zr and ^177^Lu, which were used for small-animal PET/CT imaging and TRT experiments, respectively. The DOTA to IMAB027 ratios were approximately 7.76 (Supplemental Fig. 1). Flow cytometry showed that IMAB027 did not affect cell binding when attached to DOTA (Supplemental Fig. 6). The antibody integrity of [^177^Lu]Lu-DOTA-IMAB027, IMAB027-DOTA, and IMAB027 was greater than 99%, as measured by high-performance liquid chromatography (Fig. 2A; Supplemental Figs. 2 and 3). The radiochemical purities of [^89^Zr]Zr-DFO-IMAB027 and [^177^Lu]Lu-DOTA-IMAB027 were greater than 99% (Supplemental Figs. 4 and 5). Subsequent incubation in phosphate buffered saline, normal saline (NS), and RPMI 1640 (10% fetal bovine serum) for up to 7 d resulted in no degradation of [^177^Lu]Lu-DOTA-IMAB027, as shown by radiologic thin-layer chromatography (Fig. 2B).

### Binding, Internalization, and Immunoreactive Fraction Assay

Radioligand binding assays were performed using TE-1-CLDN6, TE-15, and TE-1 cell lines. The saturation binding amount (*B*_max_) of [^177^Lu]Lu-DOTA-IMAB027 to TE-1-CLDN6 cells (1 × 10^5^) was 470.1 ± 10.56 nmol (Fig. 2C), with a half-maximal effective concentration of 1.29 ± 0.11 nmol/L and an equilibrium dissociation constant (K_d_) of 2.11 ± 0.14 nmol/L. For TE-15 cells, the B_max_ of [^177^Lu]Lu-DOTA-IMAB027 was 197.8 ± 7.72 nmol, with a K_d_ of 9.97 ± 1.4 nmol/L. Similarly, for TE-1 cells, the B_max_ was 123.0 ± 2.47 nmol, and the K_d_ was 10.92 ± 0.47 nmol/L. The results of the cell block assay showed that the radioactive uptake value of [^177^Lu]Lu-DOTA-IMAB027 in the CLDN6-positive group at 1 h was 1.25% ± 0.18% added dose/10^6^ cells (*n* = 3), whereas that in the TE-1 group and TE-1-CLDN6 blocked group was 0.47% ± 0.12% added dose/10^6^ cells and 0.14 ± 0.01% added dose/10^6^ cells (*n* = 3), respectively (Fig. 2D). The internalization and immunoreactive fraction assays were conducted using the TE-1-CLDN6 cell line. The immunoreactive fraction, determined by the Lindmo assay, was 84.6% (Fig. 2E). The internalization rate of [^177^Lu]Lu-DOTA-IMAB027 was 2.21% at 4 h and 2.32% at 24 h (Fig. 2F).

### PET/CT Imaging of [^89^Zr]Zr-DFO-IMAB027

**FIGURE 3.**
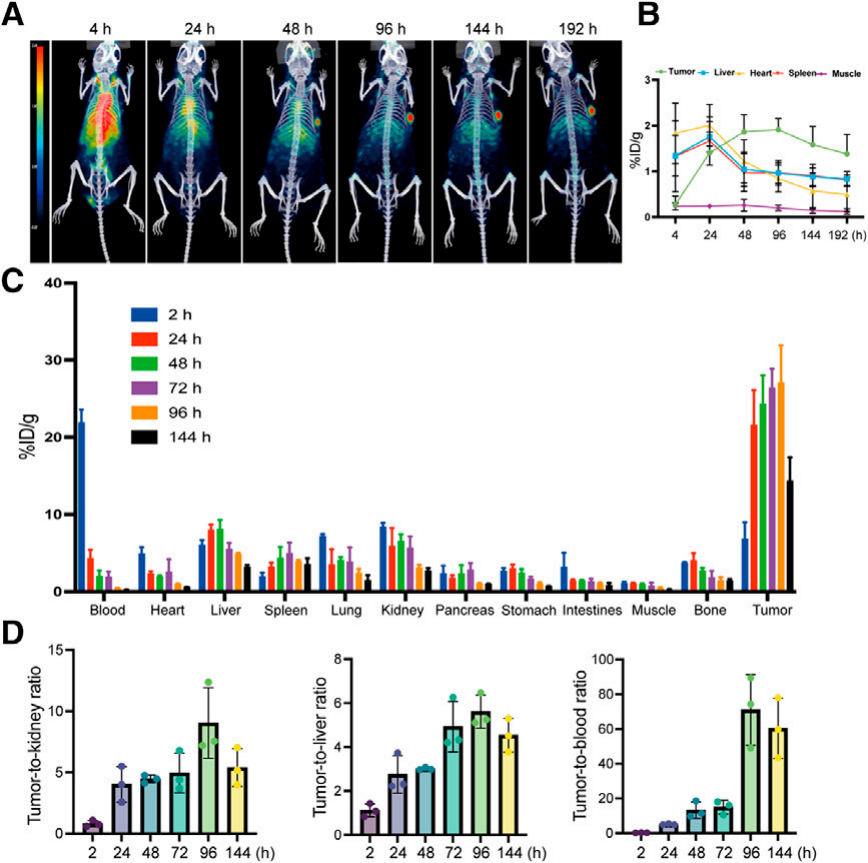
(A) PET/CT images show significant in vivo aggregation of [^89^Zr]Zr-DFO-IMAB027 in tumors of hormonal mice over time, in significant contrast to other nontarget tissues. (B) Quantitative region of interest analysis of tumor, heart, liver, and muscle in tumor-bearing mice at 4, 24, 48, 96, 144, and 192 h after injection of [^89^Zr]Zr-DFO-IMAB027. (C) Biodistribution results at 2, 24, 48, 72, 96, and 144 h after injection of [^177^Lu]Lu-DOTA-IMAB027. (D) Tumor-to-blood, tumor-to-liver, and tumor-to-kidney ratios at 2, 24, 48, 96, and 144 h after injection of [^177^Lu]Lu-DOTA-IMAB027.

PET/CT images showed that [^89^Zr]Zr-DFO-IMAB027 accumulated toward the tumor at 4 h after injection, and the tumor was clearly depicted at 24 h after injection. Radioactive uptake by the tumors gradually increased over time (Fig. 3A). We further depicted the regions of interest and quantified the radioactivity uptake in the tumor, heart (blood), liver, spleen, muscle, and kidneys (Fig. 3B). The maximum tumor uptake of [^89^Zr]Zr-DFO-IMAB027 in tumor-bearing mice occurred at 96 h, with an uptake value of approximately 26.1 ± 6.52 percent injected dose (%ID)/g (*n* = 3). Radioactivity absorption in nontarget organs such as the heart (blood), liver, and kidneys gradually decreased over time.

### Biodistribution of [^177^Lu]Lu-DOTA-IMAB027

The biodistribution results showed that [^177^Lu]Lu-DOTA-IMAB027 was targeted to the tumors of the TE-1-CLDN6 model and increased in tumor aggregation over time (Fig. 3C), with peak aggregation occurring at 96 h (27.1 ± 4.85 %ID/g). Peak uptake in the liver occurred at 48 h, with a value of 8.07 ± 1.23 %ID/g, whereas peak uptake in the kidneys occurred at 2 h, with a value of 8.35 ± 0.55 %ID/g. These values were higher than those in other nontarget tissues. Over time, uptake values in the liver and kidneys gradually decreased. [^177^Lu]Lu-DOTA-IMAB027 was rapidly cleared from the blood circulation, with blood uptake values of 21.9 ± 1.71 and 4.21 ± 1.13 %ID/g at 2 and 24 h, respectively. From 2 to 144 h after injection of [^177^Lu]Lu-DOTA-IMAB027, the tumor-to-blood ratio increased from 0.31 ± 0.08 to 67.7 ± 17.34 %ID/g, the tumor-to-liver ratio increased from 0.84 ± 0.20 to 5.61 ± 0.74 %ID/g, and the tumor-to-kidney ratio increased from 0.81 ± 0.24 to 9.04 ± 2.89 %ID/g (Fig. 3D).

### Effect of [^177^Lu]Lu-DOTA-IMAB027 Treatment

**FIGURE 4.**
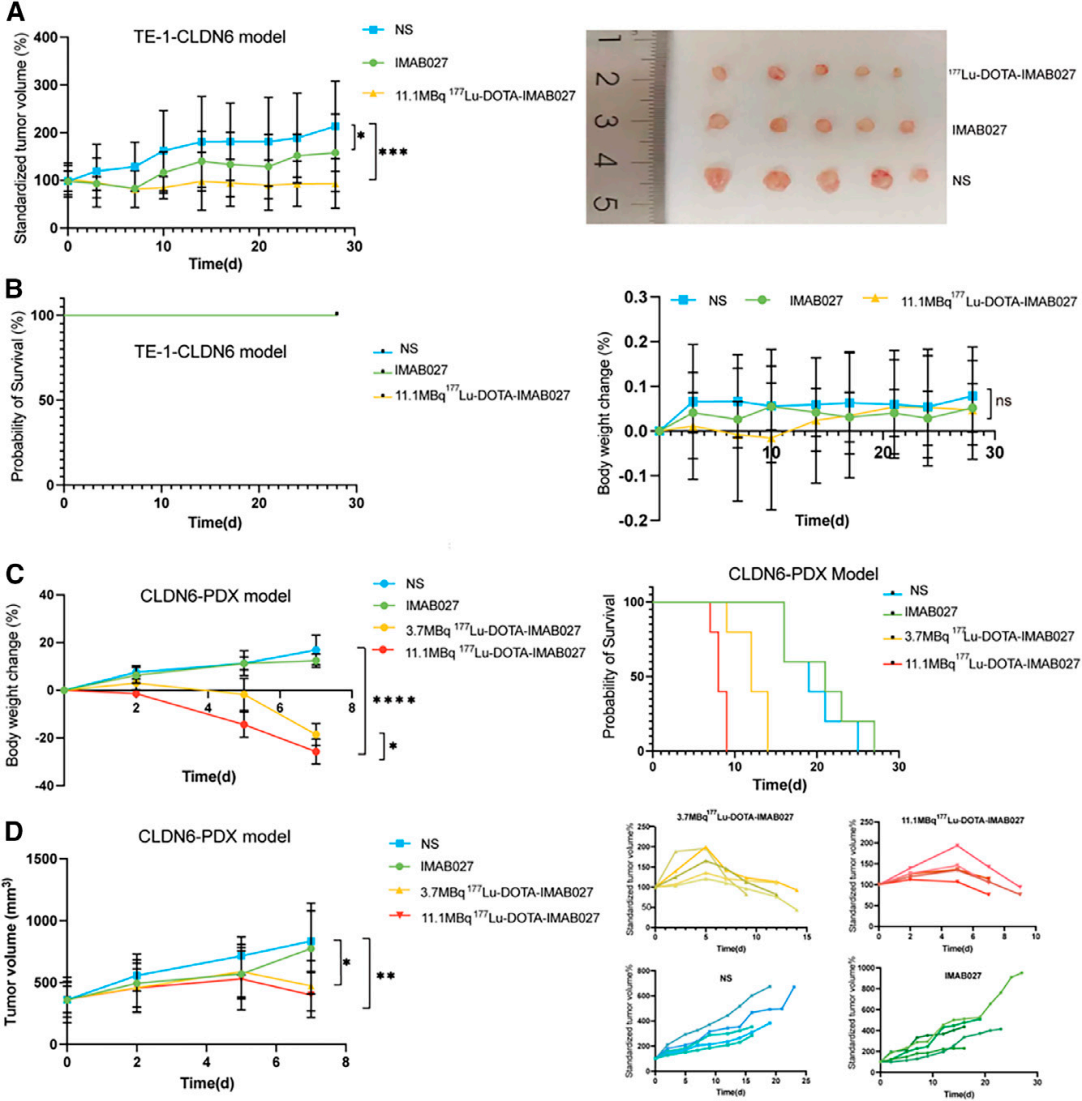
(A) Tumor growth curve of TE-1-CLDN6 model treated with 11.1 MBq of [^177^Lu]Lu-DOTA-IMAB027 or IMAB027 or NS (*n* = 5). Photos of tumor tissue in different treatment groups after treatment. (B) Body weight change curve and survival rate of TE-1-CLDN6 model. (C) Body weight change curve and survival rate of CLDN6-PDX model. (D) Tumor growth curve of CLDN6-PDX model treated with 11.1 MBq of [^177^Lu]Lu-DOTA-IMAB027, 3.7 MBq of [^177^Lu]Lu-DOTA-IMAB027, IMAB027, or NS (*n* = 5).

In the TE-1-CLDN6 model, the standardized tumor volume in the 11.1-MBq [^177^Lu]Lu-DOTA-IMAB027 group was approximately 93.46% ± 14.62% at day 28, which was significantly lower than that of the other groups (IMAB027 group: 157.88% ± 21.03%; NS group: 213.42% ± 32.06%; *P* < 0.05; Fig. 4A). The 11.1-MBq group demonstrated the strongest tumor inhibition effect, with a tumor growth inhibition percentage (TGI%) of 101.74%. The IMAB027 group exhibited a mild tumor inhibition effect, with a TGI% of 48.97%. The body weight of tumor-bearing mice in all groups showed a slow upward trend, and there was no statistically significant difference between the groups (Fig. 4B). In the patient-derived xenograft (PDX) model, on the seventh day after treatment, the standardized tumor volume in the 11.1-MBq [^177^Lu]Lu-DOTA-IMAB027 group was 108.56 ± 21.15, which was significantly lower than that in the other groups (3.7-MBq group: 129.02 ± 13.51; IMAB027 group: 231.6 ± 73.01; NS group: 244.3 ± 56.02; *P* < 0.01). The 11.1-MBq group exhibited strong tumor inhibition, with a TGI% of 79.04%. The 3.7-MBq group achieved a TGI% of 77.20%, showing a tumor inhibition effect comparable to that of the 11.1-MBq group, with no statistically significant difference between the 2 groups. The TGI% of the IMAB027 group was 12.13%, and there was no statistically significant difference in tumor volume compared with that in the NS group (Fig. 4D). The body weight of the 11.1-MBq [^177^Lu]Lu-DOTA-IMAB027 group was 15.92 ± 1.06 g, which was significantly lower than that of the 3.7-MBq [^177^Lu]Lu-DOTA-IMAB027 group, the IMAB027 group, and the NS group (Fig. 4C).

### Toxicity of [^177^Lu]Lu-DOTA-IMAB027

**FIGURE 5.**
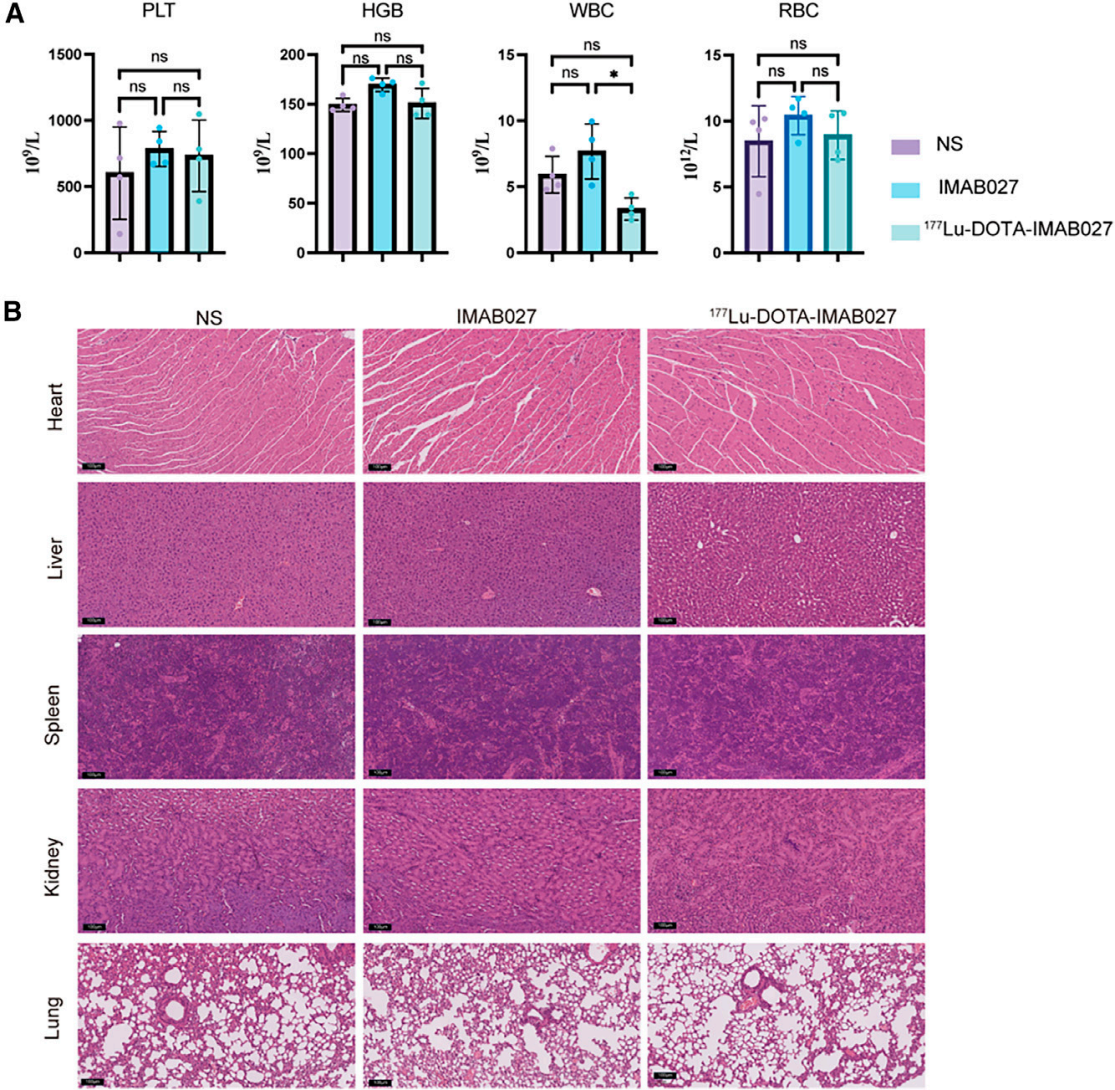
(A) WBCs, red blood cells (RBC), platelets (PLT), and hemoglobin (HGB) counts of mice in each group at 28 d after injection. (B) Representative hematoxylin and eosin staining of tissue collected from euthanized mice from [^177^Lu]Lu-DOTA-IMAB027 treatment group, IMAB027 antibody group, and NS group (*n* = 5).

White blood cell (WBC) counts after 28 d of administration in the NS group were higher than those in the [^177^Lu]Lu-DOTA-IMAB027 group but lower than those in the IMAB027 group (5.91 ± 1.33 × 10^9^/L vs. 3.32 ± 0.84 ×10^9^/L vs. 7.67 ± 2.07 × 10^9^/L). The difference in WBC counts between the [^177^Lu]Lu-DOTA-IMAB027 and IMAB027 groups was statistically significant (*P* < 0.05; Fig. 5A). We observed no statistically significant differences in the red blood cell count, platelet count, or hemoglobin levels among the groups (*P* > 0.05; Fig. 5A). Hematoxylin and eosin staining revealed no significant differences in cellular morphology or structure among the visceral organs of the [^177^Lu]Lu-DOTA-IMAB027, IMAB027, and NS groups (Fig. 5B).

**FIGURE 6.**
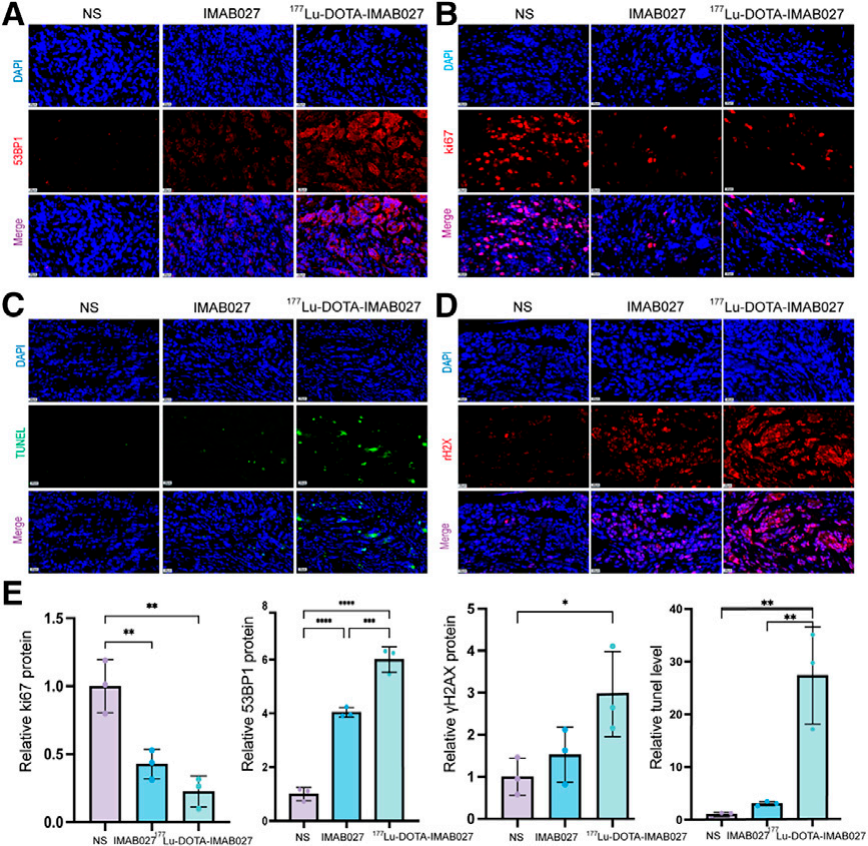
(A–D) Representative 53BP1/Ki-67/γH2Ax/TUNEL immunofluorescence staining of tumors collected from euthanized mice from [^177^Lu]Lu-DOTA-IMAB027, IMAB027, and NS groups. (E) Bar charts of relative fluorescence intensity of 53BP1/Ki-67/γH2Ax/TUNEL in each treatment group. DAPI = 4′,6-diamidino-2-phenylindole.

Immunofluorescence imaging results in tumor tissues indicated that the mean fluorescence intensity of Ki-67 in the [^177^Lu]Lu-DOTA-IMAB027 group was less than that in the NS and IMAB027 groups (*P* < 0.01; Fig. 6B). The fluorescence intensity of 53BP1 in the [^177^Lu]Lu-DOTA-IMAB027 group was higher than that in the IMAB027 and NS groups (*P* < 0.01; Fig. 6A). The fluorescence intensity of TUNEL staining in the [^177^Lu]Lu-DOTA-IMAB027 group was higher than that in the IMAB027 and NS groups (*P* < 0.01; Fig. 6C). The γH_2_AX fluorescence intensity of the [^177^Lu]Lu-DOTA-IMAB027 group was higher than that of the IMAB027 and NS groups, and the difference between the [^177^Lu]Lu-DOTA-IMAB027 and NS groups was statistically significant (*P* < 0.05; Figs. 6D and 6E).

## DISCUSSION

To the best of our knowledge, this study marks the first application of CLDN6 as a target for the treatment of EC. We confirmed that CLDN6 expression was significantly higher in EC than in normal esophageal tissue, that [^89^Zr]Zr-DFO-IMAB027 exhibited visual target specificity and sustained accumulation in CLDN6-positive EC models, and that [^177^Lu]Lu-DOTA-IMAB027 demonstrated continuous tumor suppression with controllable safety. First, the database and immunohistochemistry results both revealed elevated CLDN6 expression in EC compared with that in the normal esophagus, which is consistent with previously reported findings (13,21). Our study further revealed that approximately 79.8% of EC patients exhibit CLDN6 expression. On the basis of this result, we selected the TE-1-CLDN6 cell model, characterized by high CLDN6 expression, as well as the PDX model with high CLDN6 expression, to evaluate the antitumor efficacy of the radiopharmaceutical [^177^Lu]Lu-DOTA-IMAB027. Furthermore, since esophageal squamous cell carcinoma accounts for 85.7% of all EC cases (23), our research primarily focuses on this major subtype. Both the TE-1-CLDN6 and the PDX models used in this study represent esophageal squamous cell carcinoma to ensure that the experimental design closely aligns with clinical characteristics, thereby enhancing the potential clinical translatability of our findings.

During the experiment, the TE-1-CLDN6 model exhibited slower tumor proliferation and a longer tumor volume doubling time, resulting in relatively smaller tumor volumes during the treatment period. Despite the smaller tumor size, significant differences in tumor volumes were observed between treatment groups, demonstrating the effectiveness of the therapy. To address the limitation of small tumor volumes in this model, the PDX model experiment was initiated when tumor volumes reached 361 ± 131 mm^3^. The results showed that both 3.7-MBq and 11.1-MBq doses of [^177^Lu]Lu-DOTA-IMAB027 effectively inhibited tumor growth, with TGI% of 77.20% and 79.04%, respectively. These findings were consistent with the results of the cell line-derived xenograft (CDX) model, further supporting the antitumor efficacy of [^177^Lu]Lu-DOTA-IMAB027.

However, as the PDX model used severely immunodeficient mice that were unable to tolerate radiation therapy, the mice in the PDX treatment group began to die gradually starting from the second week of treatment. To confirm whether the deaths of NPI mice were due to their severe immunodeficiency and associated radiosensitivity, we compared the results with those of the CDX model. In the TE-1-CLDN6 model, treatment with 11.1 MBq of [^177^Lu]Lu-DOTA-IMAB027 in BALB/c nude mice resulted in no deaths or significant weight loss. Additionally, supplementary experiments were conducted in NPI mice, administering doses of 3.7, 11.1, and 14.8 MBq of [^177^Lu]Lu-DOTA-IMAB027 or 3.7 MBq of pure ^177^Lu (*n* = 3). The results showed that all NPI mice in the experimental groups died within approximately 1 wk (Supplemental Figs. 8 and 9). These findings indicate that severely immunodeficient NPI mice exhibit heightened radiosensitivity, rendering them intolerant to radionuclide-targeted therapy. Although the increased radiosensitivity of NPI mice limited further exploration of treatment-related toxicities, the results unequivocally demonstrate the significant antitumor efficacy of [^177^Lu]Lu-DOTA-IMAB027.

In our study, the antitumor activity of IMAB027 was evaluated in both CDX and PDX models. In the TE-1-CLDN6 CDX model, moderate antitumor activity was observed, which is consistent with the reported antibody-dependent cell-mediated cytotoxicity (ADCC) effects (6). However, in the PDX model established in severe immunodeficient NPI mice, no significant differences in tumor growth were observed between the IMAB027 group and the control group. The lack of significant antitumor effect in the PDX model could be attributed to the inability of NPI mice to mount an effective ADCC response, as these mice are deficient in functional B cells, T cells, and natural killer cells, all of which are critical for mediating ADCC. These results align with literature indicating that the antitumor activity of anti-CLDN6 monoclonal antibodies is primarily mediated by ADCC.

Moreover, in the TE-1-CLDN6 model, we observed the rapid clearance of [^177^Lu]Lu-DOTA-IMAB027 from the bloodstream, with an 80% reduction in unit dose between 2 and 24 h. Accelerated blood clearance minimizes blood cell damage and mitigates myelosuppression. After treatment completion, the blood cell results showed no statistically significant differences in red blood cells or platelets among the control, monoclonal antibody (mAb), and [^177^Lu]Lu-DOTA-IMAB027 groups. This is consistent with previous findings, as red blood cell and platelet counts recover after approximately 4 wk (24,25). The WBC count in the [^177^Lu]Lu-DOTA-IMAB027 group was higher than that in the control group but lower than that in the mAb group, with statistically significant differences observed only between the mAb and [^177^Lu]Lu-DOTA-IMAB027 groups. We hypothesized that this may be because the mAb stimulated a heightened immune response, leading to an increase in WBCs, whereas antibodies in [^177^Lu]Lu-DOTA-IMAB027 similarly stimulated elevated WBCs, although circulating ^177^Lu may induce WBC damage. Hematoxylin and eosin staining of the major organs showed no significant differences among the control, mAb, and [^177^Lu]Lu-DOTA-IMAB027 groups. Furthermore, according to hematologic analysis and body weight results, [^177^Lu]Lu-DOTA-IMAB027 inhibited tumor growth and exhibited no significant blood and organ toxicity.

Additionally, although we primarily focused on CLDN6 as a target for the imaging and treatment of EC, CLDN6 is upregulated in many common cancers, such as ovarian, breast, and gastric cancers (26–29). Therefore, this therapeutic probe has potential applications in various tumors with high CLDN6 expression. In summary, we propose [^177^Lu]Lu-DOTA-IMAB027 as a novel drug for treating patients with advanced metastatic lesions and potential metastases.

## CONCLUSION

CLDN6 expression was upregulated in esophageal carcinoma. The novel radiotracers [^89^Zr]Zr-DFO-IMAB027 and [^177^Lu]Lu-DOTA-IMAB027 achieved the noninvasive assessment of CLDN6 expression and inhibited tumor growth in EC with high CLDN6 expression, respectively. Thus, the CLDN6-targeted radioligand diagnosis and treatment of EC is a promising method that warrants further clinical research.

## DISCLOSURE

This study was financially supported by the Key Laboratory of Nuclear Technology and Medical Transformation of the National Health Commission (Nos. 2021HYX015 and 2022HYX015) and INPC Innovative Talents Training Program (No. YC0701). No other potential conflict of interest relevant to this article was reported.

## Notes

KEY POINTS **QUESTION:** Is CLDN6 a useful target for the imaging and radiopharmaceutical therapy of EC using a novel pair of radioactive nuclides, ^89^Zr and ^177^Lu? **PERTINENT FINDINGS:** The differential expression of CLDN6 between tumors and the normal esophagus highlights its potential as a diagnostic and therapeutic target for EC. [^89^Zr]Zr-DFO-IMAB027 showed high contrast when visualizing CLDN6-expressing xenografts for PET imaging, and [^177^Lu]Lu-DOTA-IMAB027 induced rapid tumor regression in a preclinical model of EC. **IMPLICATIONS FOR PATIENT CARE:** Using CLDN6-targeted radioligand diagnosis and treatment of EC is a promising method that warrants further clinical research.
